# 抑制肺鳞癌靶点HMGCS1促进细胞铁死亡

**DOI:** 10.3779/j.issn.1009-3419.2024.101.12

**Published:** 2024-05-20

**Authors:** NI Yinyun, YANG Ying, ZHANG Li

**Affiliations:** 610000 成都，四川大学华西医院疾病分子网络前沿科学中心呼吸健康研究所; Institute of Respiratory Health, Frontiers Science Center for Disease-related Molecular Network, West China Hospital, Sichuan University, Chengdu 610000, China

**Keywords:** 肺肿瘤, HMGCS1, 铁死亡, Hymeglusin, Lung neoplasms, HMGCS1, Ferroptosis, Hymeglusin

## Abstract

**背景与目的:**

靶向治疗在肺鳞癌中的效果不理想，而免疫治疗较低的应答率阻碍了其在肺鳞癌中的应用，因此，探索肺鳞癌治疗的新策略十分迫切。铁死亡在抑制肿瘤方面发挥了重要作用，本研究旨在探究靶向3-羟基-3-甲基戊二酰辅酶A合酶1（3-hydroxy-3-methylglutaryl-CoA synthase 1, HMGCS1）对肺鳞癌细胞铁死亡的调控和作用机制，为肺鳞癌治疗提供新的研究思路和方向。

**方法:**

通过肿瘤基因组图谱（The Cancer Genome Atlas, TCGA）和临床蛋白质组肿瘤分析联盟（Clinical Proteomic Tumor Analysis Consortium, CPTAC）在线蛋白数据库分析HMGCS1在肺鳞癌中的表达情况；通过Kaplan-Meier Plotter在线生存数据库分析HMGCS1与肺癌生存时间的关系；通过免疫组化验证HMGCS1在肺鳞癌组织的表达水平；在肺鳞癌细胞系SKMES细胞通过小干扰RNA（small interfering RNA, siRNA）干扰HMGCS1表达后，通过CCK8和Transwell检测细胞活性和细胞迁移能力；通过流式细胞术检测干扰HMGCS1后或用HMGCS1抑制剂hymeglusin处理后细胞凋亡水平；通过流式和高内涵共聚焦荧光成像系统分别检测抑制HMGCS1后SKMES细胞中Fe^2+^、活性氧（reactive oxygen species, ROS）和脂质过氧化水平；通过Western blot检测抑制HMGCS1后铁死亡通路标志蛋白ACSL4、GPX4和SLC7A11表达。

**结果:**

HMGCS1 mRNA和蛋白水平在肺鳞癌均显著高表达；siRNA干扰HMGCS1表达抑制了肺鳞癌细胞增殖活性和迁移能力，但对细胞凋亡没有显著影响。干扰HMGCS1后或用HMGCS1抑制剂hymeglusin处理后显著促进了SKMES细胞内Fe^2+^、ROS和脂质过氧化水平，促进肺鳞癌细胞铁死亡；Western blot检测显示抑制HMGCS1显著促进了ACSL4的表达。

**结论:**

抑制肺鳞癌靶点HMGCS1可促进肺癌细胞铁死亡，为肺鳞癌筛选新的治疗靶点提供了研究基础。

## Abstract (en)

**Background and objective:**

Targeted therapies are ineffective in lung squamous cancer (LUSC), and the low response rate of immunotherapy hampers its application in LUSC, so it is urgent to explore new strategies for LUSC treatment. Ferroptosis plays an important role in tumour suppression. The aim of this study was to investigate the role and mechanism of targeting 3-hydroxy-3-methylglutaryl-CoA synthase 1 (HMGCS1) in regulating ferroptosis in LUSC cells, in order to provide a new research direction for LUSC therapy.

**Methods:**

The expression of HMGCS1 in LUSC was analysed by The Cancer Genome Atlas (TCGA) and Clinical Proteomic Tumor Analysis Consortium (CPTAC) online databases; the relationship between HMGCS1 and survival time of lung cancer was analysed by the Kaplan-Meier Plotter online survival database; the expression level of HMGCS1 in LUSC tissues was verified by immunohistochemistry. After interfering with HMGCS1 expression by small interfering RNA (siRNA), cell activity and cell migration ability were detected by CCK8 and Transwell assay; apoptosis was detected by flow cytometry after interfering with HMGCS1 or after treatment with the HMGCS1 inhibitor of hymeglusin; Fe^2+^, reactive oxygen species (ROS) and lipid peroxidation levels were detected by flow cytometry and high-content confocal fluorescence imaging systems, respectively in SKMES cells after inhibition of HMGCS1; and Western blot was performed to detect the expression of ACSL4, GPX4 and SLC7A11, which are markers of the ferroptosis pathway after inhibition of HMGCS1.

**Results:**

HMGCS1 mRNA and protein levels were significantly high in LUSC; siRNA interference with HMGCS1 expression inhibited the proliferative activity and migration ability of LUSC cells, but had no significant effect on apoptosis. Interference with HMGCS1 or treatment with the HMGCS1 inhibitor of hymeglusin significantly promoted intracellular Fe^2+^, ROS and lipid peroxidation levels in SKMES cells, and induced ferroptosis in LUSC cells; Western blot assay showed that inhibition of HMGCS1 significantly promoted the expression of ACSL4.

**Conclusion:**

Inhibition of HMGCS1, a target of LUSC, promotes ferroptosis in lung cancer cells and provides a research basis for screening new therapeutic targets for LUSC.

在全球范围内，肺癌仍然是发病率和致死率极高的恶性肿瘤之一^[1]^。其中，非小细胞肺癌占所有肺癌病例的85%以上，肺腺癌和肺鳞癌是非小细胞肺癌最主要的两种亚型^[2]^。而肺鳞癌占非小细胞肺癌的25%-30%，5年生存率低于20%，每年约有40万人因其死亡^[3]^。与肺腺癌不同，肺鳞癌中表皮生长因子受体（epidermal growth factor receptor, EGFR）突变率较低，因此作为非小细胞肺癌一线治疗的EGFR酪氨酸激酶抑制剂在肺鳞癌中的效果不理想^[4]^。尽管肺鳞癌对普通辅助疗法反应不佳，但免疫检查点抑制剂疗法在肺鳞癌中显示出令人振奋的疗效，目前已有多种药物应用于肺鳞癌治疗^[5]^。然而，由于肿瘤的异质性、肿瘤微环境的复杂性以及不同的免疫浸润模式，相对较低的应答率和严重的不良反应仍阻碍了免疫治疗在肺鳞癌患者中的应用^[6]^。因此，探索肺鳞癌治疗的新策略十分迫切。

铁死亡是一种铁依赖的由脂质过氧化驱动的、有别于细胞凋亡的独特细胞程序性死亡方式，在抑制肿瘤方面发挥了重要作用^[7]^。研究显示，诱导铁死亡是杀死对常规治疗产生抗药性的癌细胞的有效手段，如增强化疗^[8]^和放疗^[9]^效果等。此外肿瘤免疫治疗方面，铁死亡诱导剂和细胞程序性死亡配体1（programmed cell death ligand 1, PD-L1）阻断剂能够协同增强临床前模型的抗肿瘤活性^[10]^。因此研究肺鳞癌的铁死亡诱导靶点将为肺鳞癌治疗提供新的研究方向。铁死亡执行的关键是铁催化的底物——包括多不饱和脂肪酸的磷脂 ( polyunsaturated fatty acid binding phospholipids, PUFA-PLs）的过氧化，脂质过氧化物在细胞膜上的过度积累会导致细胞膜破裂，从而导致细胞的铁死亡^[11]^。乙酰辅酶A合成酶长链家族成员4（acyl-CoA synthetase long-chain family member 4, ACSL4）是PUFA-PL合成的关键调控因子，可以促进脂质过氧化的生成，从而导致铁死亡^[12]^。

3-羟基-3-甲基戊二酰辅酶A合酶1（3-hydroxy-3-methylglutaryl-CoA synthase 1, HMGCS1）是甲羟戊酸途径中的限速酶，可以催化HMG-CoA的合成^[13]^。HMGCS1在人类肿瘤中的功能研究较少，尤其在肺鳞癌中的表达和功能仍然未知。本研究探讨了靶向HMGCS1对肺鳞癌铁死亡的调控作用，同时发现其抑制剂hymeglusin也可以促进肺鳞癌细胞铁死亡。进一步机制研究发现抑制HMGCS1通过促进ACSL4表达诱导肺癌细胞铁死亡，为肺鳞癌筛选新的治疗靶点提供了研究基础。

## 1 材料和方法

### 1.1 材料

肺鳞癌细胞系SKMES细胞购买自中科院细胞库。用含有10%血清的MEM培养基，添加1% Glutamax、1%非必需氨基酸溶液（100×）和1%丙酮酸钠溶液（100 mmol/L），在37 °C、5% CO_2_孵箱培养。HMGCS1（#17643-1-AP）和ACSL4（#22401-1-AP）抗体购自Proteintech，GPX4（#52455S）和SLC7A11（#12691S）抗体购自CST，GAPDH 抗体购自Boster（#BA2913）。

### 1.2 免疫组织化学染色

将脱蜡后的切片依次进行如下处理：（1）微波高温抗原修复；（2）3% H_2_O_2_封闭；（3）加一抗，4 °C过夜；（4）加二抗，37 °C孵箱中孵育30 min；（5）DAB显色；（6）复染：用水冲洗后用苏木精染色30 s；（7）脱水：用水冲洗后，用梯度酒精脱水浸泡于二甲苯中；（8）封片晾干。

### 1.3 小干扰RNA（small interfering RNA, siRNA）干扰

HMGCS1 siRNA购自汉恒公司，序列为：NC-UUCUCCGAACGUGUCACGUTT；HMGCS1-si-GGAUGUUGCUGAAUGACUU，在转染前一天接种1.5×10^5^个SKMES细胞于6孔板中，24 h后，用jetPRIME转染试剂转染。根据转染试剂说明书，用250 µL jetPRIME转染buffer稀释10 µL siRNA（20 μmol/L储存液），并用枪轻轻吹打3-5次混匀，再加入4 µL jetPRIME转染试剂，用枪轻轻吹打3-5次混匀，室温孵育10 min，均匀加入到细胞板中，前后轻摇细胞板混合均匀。

### 1.4 Western blot检测

将不同组细胞进行蛋白质提取后用BCA法测定蛋白质浓度，将蛋白用1×loading buffer重悬后100 °C煮沸5-10 min进行蛋白质变性，每组细胞按30 μg上样，分别经过电泳、转膜、封闭、一抗4 °C孵育过夜后进行二抗孵育，最后显影。

### 1.5 CCK8和Transwell检测

将不同组对数生长期细胞以（3-5）×10^3^/孔培养于96孔板（100 μL/孔），药物处理72 h后添加CCK-8检测试剂用酶标仪检测450 nm波长下吸光度。用Transwell小室检测细胞迁移能力，在小室上方铺（2-3）×10^4^细胞（200 μL/孔），48 h后用结晶紫染色。

### 1.6 Fe^2+^检测

加入浓度为1 μmol/L的FerroOrange工作液（DOJINDO），在37 °C、5% CO_2_培养箱中孵育30 min，用流式检测或高内涵采图，Ex：561 nm/Em：570-620 nm。

### 1.7 活性氧（reactive oxygen species, ROS）和脂质过氧化检测

收集不同处理组细胞用PBS洗涤细胞1次，分别加入2.5 μmol/L CellROX^TM^ Deep Red，2.5 μmol/L BODIPY 581/591 C11工作液，在37 °C、5% CO_2_培养箱中孵育30 min，用PBS清洗1次后用流式检测或高内涵采图。

### 1.8 统计学方法

数据使用Graph Prism 8.0软件进行分析，所有实验至少进行了3次独立实验，结果以均数±标准差表示，并显示了具有代表性的图像。两组数据比较采用t检验，P<0.05被认为差异具有统计学意义。

## 2 结果

### 2.1 HMGCS1在肺鳞癌组织高表达

为了分析HMGCS1在肺癌组织中的表达情况，我们通过UALCAN在线数据库中的肿瘤基因组图谱（The Cancer Genome Atlas, TCGA）和临床蛋白质组肿瘤分析联盟（Clinical Proteomic Tumor Analysis Consortium, CPTAC）数据，发现相比正常对照组，HMGCS1 mRNA和蛋白水平在肺鳞癌样本均显著高表达（图1A、1B）。为了进一步分析HMGCS1与肺癌患者生存时间的关系，我们通过Kaplan-Meier Plotter在线生存分析中的肺癌数据集分析显示，在2166例肺癌患者中，HMGCS1高表达和肺癌患者的生存时间降低显著相关。低表达组的中位生存时间为86个月，而高表达组的中位生存时间仅为55个月（图1C）。此外，我们通过免疫组化验证了HMGCS1在肺鳞癌组织中的表达水平，发现在10例肺正常组织中，HMGCS1表达均为阴性，而在25例肺鳞癌组织中，HMGCS1阴性或弱阳表达的组织有7例，占28%，而强阳和极强阳表达的组织分别为10和8例，总占比为72%（图1D）。以上结果表明，HMGCS1在肺鳞癌组织中高表达，并且其高表达与肺癌患者较短的生存时间相关。

**图 1:**
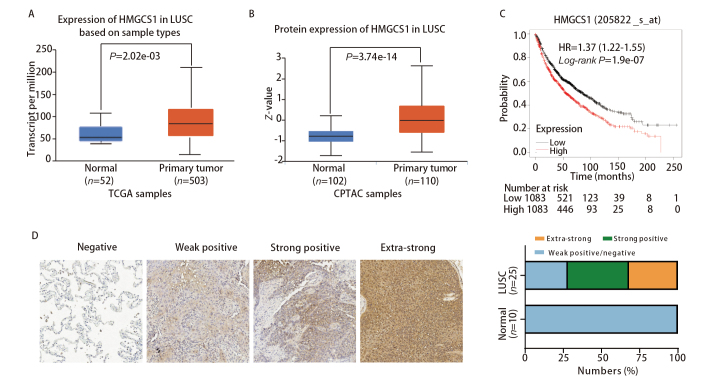
HMGCS1在肺鳞癌组织高表达。A：UALCAN在线数据库中的TCGA数据分析显示HMGCS1 mRNA水平在肺鳞癌组织中高表达；B：UALCAN在线数据库中的CPTAC数据分析显示HMGCS1蛋白水平在肺鳞癌组织中高表达；C：Kaplan-Meier Plotter在线生存分析显示HMGCS1的表达和肺癌生存时间相关；D：免疫组化验证HMGCS1在肺正常组织（n=10）和肺鳞癌组织（n=25）中的表达水平（×30）。

### 2.2 敲低HMGCS1抑制肺鳞癌细胞增殖和迁移

为了研究HMGCS1对肺鳞癌细胞功能的影响，我们在肺鳞癌细胞SKMES中通过siRNA抑制了HMGCS1表达，Western blot结果显示HMGCS1 siRNA显著抑制了其在SKMES细胞中的蛋白表达水平（图2A）。接着我们通过CCK8检测了抑制HMGCS1后的细胞增殖活性，结果显示，和对照组相比，干扰HMGCS1后SKMES细胞活性显著降低（图2B）。同时收集干扰HMGCS1 24 h后的细胞铺至Transwell小室进行细胞迁移能力检测，48 h后通过结晶紫染色发现抑制HMGCS1显著抑制了肺鳞癌细胞迁移能力（图2C、2D）。我们进一步通过流式细胞术检测了抑制HMGCS1后对细胞凋亡的影响，结果显示敲低HMGCS1对肺鳞癌细胞的凋亡和对照组相比没有显著差异（图2E、2F）。以上结果表明敲低HMGCS1抑制肺癌细胞增殖和迁移，但对细胞凋亡没有显著影响，可能存在其他调控作用。

**图 2:**
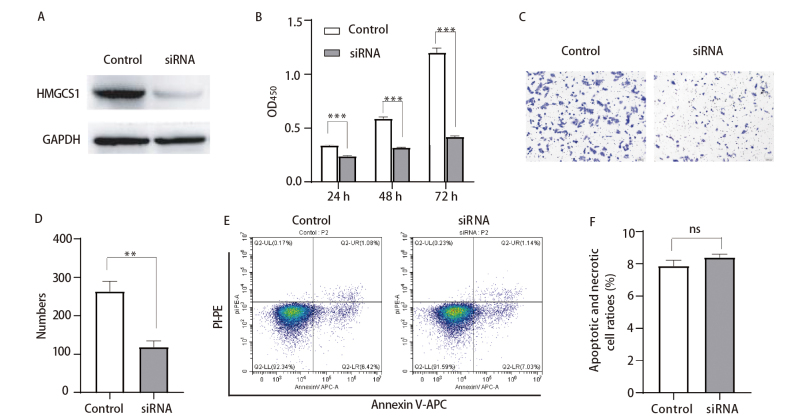
敲低HMGCS1抑制肺鳞癌细胞增殖和迁移。A：Western blot验证HMGCS1 siRNA干扰效果；B：CCK8检测抑制HMGCS1后细胞活性；C：Transwell检测抑制HMGCS1后细胞迁移能力（×20）；D：细胞迁移计数统计；E：流式细胞术检测抑制HMGCS1后细胞凋亡水平；F：凋亡细胞百分比统计。**P<0.01；***P<0.001。

### 2.3 敲低HMGCS1诱导肺鳞癌细胞发生铁死亡

由于HMGCS1参与的甲羟戊酸途径与铁死亡密切相关^[14]^，为了进一步研究HMGCS1和铁死亡的调控关系，我们分别用Ferroorange、CellROX和BODIPY C11进行细胞中Fe^2+^、ROS和脂质过氧化染色，并通过流式和高内涵荧光成像系统进行检测。结果显示抑制HMGCS1表达后显著促进了细胞内Fe^2+^（图3A、3B）、ROS（图3C、3D）和脂质过氧化水平（图3E、3F），诱导肺癌细胞发生铁死亡。以上结果表明抑制HMGCS1诱导肺鳞癌细胞发生铁死亡。

**图 3:**
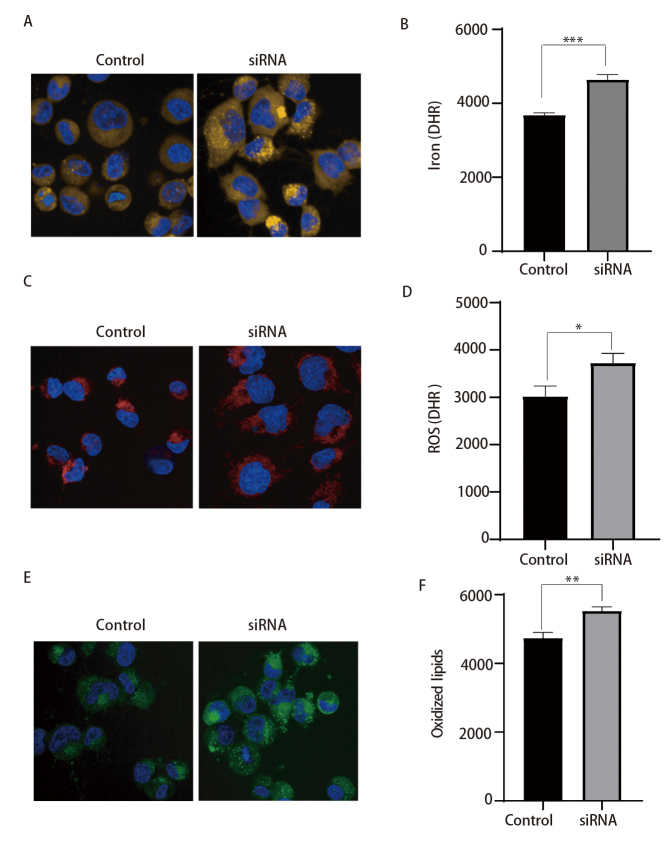
敲低HMGCS1诱导肺鳞癌细胞发生铁死亡。A：高内涵荧光成像检测抑制HMGCS1后Fe^2+^水平（×200）；B：流式检测抑制HMGCS1后Fe^2+^水平；C：高内涵荧光成像检测抑制HMGCS1后ROS水平（×200）；D：流式检测抑制HMGCS1后ROS水平；E：高内涵荧光成像检测抑制HMGCS1后脂质过氧化水平（×200）；F：流式检测抑制HMGCS1后脂质过氧化水平。*P<0.05；**P<0.01；***P<0.001。

### 2.4 HMGCS1抑制剂hymeglusin促进肺鳞癌细胞铁死亡

为了研究HMGCS1抑制剂hymeglusin是否可以同样抑制肺鳞癌细胞增殖，我们用不同浓度的hymeglusin处理SKMES细胞和肺正常上皮细胞BEAS，结果发现hymeglusin呈剂量依赖方式抑制了SKMES细胞活性，而对BEAS在初始浓度有一定抑制，但随着浓度加大对细胞活性的影响趋于稳定（图4A），表明肺鳞癌细胞SKMES相比正常肺上皮细胞对hymeglusin更敏感。我们进一步检测了hymeglusin对细胞凋亡的影响，虽然hymeglusin对SKMES细胞凋亡有一定的促进作用，但仅从约2%提高到了3.5%左右，表明对细胞凋亡的影响较小（图4B、4C）。接着为了研究hymeglusin对肺鳞癌细胞铁死亡的作用，我们用Ferroorange、CellROX和BODIPY C11进行细胞内Fe^2+^、ROS和脂质过氧化染色，并通过高内涵荧光成像系统进行检测。结果显示用hymeglusin处理后显著促进了细胞内Fe^2+^（图4D、4E）、ROS（图4F、4G）和脂质过氧化水平（图4H、4I），同样诱导肺癌细胞发生了铁死亡。最后，为了探索抑制HMGCS1促进铁死亡的机制，我们通过Western blot检测了铁死亡通路经典标志蛋白的表达，包括ACSL4、GPX4和SLC7A11，结果显示抑制HMGCS1或用hymeglusin处理SKMES细胞，GPX4和SLC7A11的表达没有显著变化，而ACSL4的表达却显著升高（图4J）。以上结果表明抑制HMGCS1主要通过促进ACSL4表达诱导肺鳞癌细胞发生铁死亡。

**图 4:**
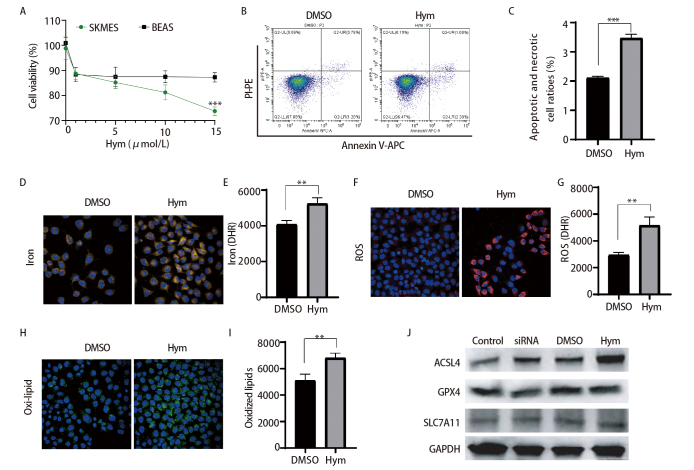
Hymeglusin促进肺鳞癌细胞铁死亡。A：CCK8检测不同浓度hymeglusin对SKMES和BEAS的抑制作用；B：hymeglusin (15 μmol/L) 作用SKMES细胞48 h后细胞凋亡检测；C：凋亡细胞百分比统计；D：高内涵荧光成像检测hymeglusin (15 μmol/L) 作用SKMES细胞48 h后Fe^2+^水平（×80）；E：Fe^2+^荧光强度统计；F：高内涵荧光成像检测hymeglusin (15 μmol/L) 作用SKMES细胞48 h后ROS水平（×80）；G：ROS荧光强度统计；H：高内涵荧光成像检测hymeglusin (15 μmol/L) 作用SKMES细胞48 h后脂质过氧化水平（×80）；I：脂质过氧化荧光强度统计；J：Western blot检测ACSL4、GPX4和SLC7A11蛋白表达，GAPDH为内参。**P<0.01；***P<0.001。

## 3 讨论

肺癌是癌症相关死亡的主要原因^[1,3]^。与肺腺癌不同，肺鳞癌患者并未从靶向治疗中获益^[4,15]^。迄今为止，只有免疫疗法已发展成为肺鳞癌患者的成功治疗策略^[5,16]^，然而较低的应答率和严重的不良反应阻碍了免疫治疗在肺鳞癌患者中的应用^[6]^，因此探索肺鳞癌治疗的新策略十分迫切。本研究发现代谢酶HMGCS1在肺鳞癌组织中高表达，通过siRNA抑制HMGCS1表达后显著诱导了肺鳞癌SKMES细胞Fe^2+^、ROS和脂质过氧化水平，表明靶向HMGCS1可以诱导肺癌细胞发生铁死亡。进一步在体外用HMGCS1抑制剂hymeglusin处理SKMES细胞后，同样诱导肺癌细胞发生铁死亡。

作为甲羟戊酸合成途径中的关键酶之一，HMGCS1催化乙酰乙酰辅酶A向HMG-CoA的转化，从而在甲羟戊酸代谢中发挥关键作用^[17]^。研究^[18]^显示在多种癌症类型中都观察到HMGCS1表达升高，并与生存时间密切相关。在本研究中，我们同样发现HMGCS1在肺鳞癌高表达，其高表达与肺癌生存时间呈显著负相关。甲羟戊酸途径的代谢物，如角鲨烯和胆固醇等，在多个水平上参与了对铁死亡的调控^[14]^。Zhou等^[19]^发现脯氨酰4-羟化酶（prolyl-4-hydroxylase, P4H）亚基P4HA1可通过激活甲羟戊酸通路中的关键酶HMGCS1保护鼻咽癌细胞免受erastin诱导的铁死亡。然而HMGCS1在肺鳞癌中的功能尚未见报道。在本研究中，我们发现靶向抑制肺鳞癌细胞HMGCS1表达后，细胞增殖和迁移能力受到抑制，同时细胞Fe^2+^、ROS和脂质过氧化水平均显著升高。同时用HMGCS1抑制剂hymeglusin处理SKMES细胞后，同样诱导细胞Fe^2+^、ROS和脂质过氧化水平显著升高，表明抑制HMGCS1诱导了肺鳞癌细胞发生铁死亡。

铁死亡通过毒性的脂质过氧化的积累触发，是一种铁离子依赖型的细胞死亡方式^[7]^。其已知的经典信号调控通路由谷胱甘肽过氧化物酶4（glutathione peroxidase 4, GPX4）介导。溶质载体家族7成员11（SLC7A11；也称为xCT）是系统xc-中的转运蛋白亚基。用胱氨酸缺乏培养基或通过erastin或者其他FIN药物阻断SLC7A11介导的胱氨酸转运能够在许多类型的癌症细胞中诱导铁死亡。SLC7A11-谷胱甘肽-GPX4轴被认为构成了抵抗铁死亡的主要调节机制^[11]^。因此我们进一步通过Western blot检测了抑制HMGCS1后GPX4和SLC7A11的表达，发现对GPX4和SLC7A11均没有显著的抑制作用，而对ACSL4有显著的上调作用。研究^[11,20]^表明，ACSL4依赖性磷脂的调节是对铁死亡敏感性的关键决定因素。因此靶向HMGCS1可能主要通过促进ACSL4表达诱导铁死亡。

但本研究仍具有一定局限性，一方面仅有体外细胞实验，没有研究体内HMGCS1对肺鳞癌进程的作用，无法有效反映体内微环境的复杂性；另一方面在机制研究中没有更深入地正反论证ACLS4介导的作用，在今后研究中需要进一步探索。综上所述，本研究发现了肺鳞癌新的铁死亡靶点HMGCS1，为肺鳞癌治疗策略提供了新的研究方向。
